# Use of sodium bicarbonate in out-of-hospital cardiac arrest: a systematic review and meta-analysis

**DOI:** 10.1186/s12245-021-00344-x

**Published:** 2021-04-13

**Authors:** Mohammed S. Alshahrani, Hassan W. Aldandan

**Affiliations:** 1 Department of emergency and critical care medicine, College of Medicine, Imam Abdulrahman Bin Faisal University, Alkhobar, Saudi Arabia; 2 Department of Critical Care Medicine, King Fahad Hospital of the University, Alkhobar, Saudi Arabia

**Keywords:** Sodium bicarbonate, Out-of-hospital cardiac arrest, Meta-analysis

## Abstract

**Background:**

Out-of-hospital cardiac arrest (OHCA) is a common cause of death worldwide (Neumar et al., Circulation 122:S729–S767, 2010), affecting about 300,000 persons in the USA on an annual basis; 92% of them die (Roger et al., Circulation 123:e18–e209, 2011). The existing evidence about the use of sodium bicarbonate (SB) for the treatment of cardiac arrest is controversial. We performed this study to summarize the evidence about the use of SB in patients with out-of-hospital cardiac arrest (OHCA).

**Methods:**

We searched PubMed, Scopus, EBSCO, Web of Science, and Cochrane Library, until June 2019, for randomized controlled trials (RCTs) and observational studies that used SB in patients with OHCA. Outcomes of interest were the rate of survival to discharge, return of spontaneous circulation (ROSC), sustained ROSC, and good neurological outcomes at discharge. Odds ratio (OR) with their 95% confidence interval (CI) were pooled in a random or fixed meta-analysis model.

**Results:**

A total of 14 studies (four RCTs and 10 observational studies) enrolling 28,412 patients were included; of them, eight studies were included in the meta-analysis. The overall pooled estimate did not favor SB or control in terms of survival rate at discharge (OR= 0.66, 95% CI [0.18, 2.44], *p*=0.53) and ROSC rate (OR= 1.54, 95% CI [0.38, 6.27], *p*=0.54), while the pooled estimate of two studies showed that SB was associated with less sustained ROSC (OR= 0.27, 95% CI [0.07, 0.98], *p*=0.045) and good neurological outcomes at discharge (OR= 0.12, 95% CI [0.09, 0.15], *p*<0.01).

**Conclusion:**

The current evidence demonstrated that SB was not superior to the control group in terms of survival to discharge and return of spontaneous circulation. Further, SB was associated with lower rates of sustained ROSC and good neurological outcomes.

**Supplementary Information:**

The online version contains supplementary material available at 10.1186/s12245-021-00344-x.

## Introduction

Out-of-hospital cardiac arrest (OHCA) is a common cause of death worldwide [1], affecting about 300,000 persons in the USA on an annual basis; 92% of them die [2]. An OHCA is defined as cessation of the mechanical activity of the heart outside the hospital with absence of circulation signs. Most OHCA events are of cardiac origin (70 to 85%), while some cases occur due to non-cardiac causes, such as posttraumatic, drug overdose, drowning, primary respiratory arrest, electrocution, and asphyxia [3].

In an attempt to restore cardiopulmonary functions and achieve good neurological outcomes, the advanced life support (ALS) guidelines have been extensively developed and applied internationally. Sodium bicarbonate (SB) has been routinely used during cardiopulmonary resuscitation (CPR) in cardiac arrest to correct metabolic acidosis. Earlier ALS guidelines have recommended the use of SB in cardiac arrest, and it was frequently used until the mid-1980s [4, 5]. However, the American Heart Association (AHA) guidelines in 1986 raised many doubts regarding the safety and efficacy of SB [6]. The following guidelines in 1992 [7] and 2000 [8] discouraged its use. Moreover, SB had been shown to cause hypernatremia, alkalosis, and excess carbon dioxide during CPR [9]. For these reasons, the updated AHA guidelines in 2010 did not recommend SB administration in cardiac arrest patients [1], except for hyperkalemia or tricyclic antidepressant overdose or cases of severe cardiotoxicity [10, 11].

The existing evidence about the use of SB for the treatment of cardiac arrest is controversial. A previous study suggested that early and more frequent use of SB resulted in favorable short and long outcomes [12]. Another recent study showed that SB use led to an increase in return of spontaneous circulation (ROSC) rate [13]. On the other hand, other reports failed to establish beneficial effects of SB in cardiac arrest [14–16].

In this systematic review and meta-analysis, we aimed to assess the effect of SB on the survival rate to hospital discharge, ROSC, and good neurological outcomes at discharge in patients with OHCA.

## Methods

We performed all steps of this systematic review in strict accordance with Cochrane Handbook of Systematic Reviews and Meta-analysis [17]. We also followed the preferred reporting items for systematic reviews and meta-analyses (PRISMA statement guidelines) during drafting our manuscript [18].

### Literature search

We searched PubMed, Scopus, EBSCO, Web of Science, and the Cochrane Central Register of controlled trials (CENTRAL) to identify relevant studies, until June 2019, without any language restrictions. The following search queries were used independently or in combination according to the medical subject headings (MESH) (“sodium bicarbonate”, “bicarbonate”, “NaHCO3, “cardiac arrest”, and “cardiopulmonary resuscitation”). We searched ClinicalTrials.gov to identify additional relevant studies. Furthermore, we hand-searched references of the most relevant articles.

### Eligibility criteria

We included studies that met the following criteria:

- Study design: randomized controlled trials (RCTs) and observational studies.
- Population: adult patients with OHCA.
- Intervention: SB administration during cardiopulmonary resuscitation.
- Comparator: no administration of SB during cardiopulmonary resuscitation.
- Outcomes: primary outcome measured was the survival rate to hospital discharge and secondary outcomes were ROSC, sustained ROSC defined as the restoration of a palpable pulse ≥20 min, and good neurological outcome at discharge defined as cerebral performance category (CPC) 1 or 2 or modified Rankin scale of 3 or less.

We excluded animal studies, reviews, case reports, case series, conference abstracts, and duplicate references.

### Study selection

Two authors independently applied the selection criteria. Eligibility screening was conducted in two steps: (a) title and abstract screening for matching the inclusion criteria and (b) full-text screening for eligibility to meta-analysis using a standardized Excel spreadsheet.

### Data extraction

Data extraction was carried out by two researchers, while a third researcher resolved any disputes between the two main researchers. The data collected were the first author’s name, publication year, country, study design, interventions, number of participants, age, gender, study period, and outcomes of interest.

### Risk of bias assessment

We used the Cochrane risk of bias (ROB) assessment tool [19], which includes five types of bias: selection bias (sequence generation and allocation concealment), performance bias (blinding of participants and investigators), detection bias (blinding of outcome assessors), attrition bias (incomplete outcome data), and reporting bias (selective outcome reporting). Each study is classified in each domain as low, high, or unclear risk of bias. We evaluated the quality of observational studies using the Newcastle Ottawa scale (NOS) [20], which assesses studies based on three domains: (a) selection of the study subjects, (b) comparability of groups on demographic characteristics and important potential confounders, and (c) ascertainment of the prespecified outcome (exposure/treatment).

### Data analysis and assessment of heterogeneity

The analyses were performed using the R software for Windows (version 3.5-3, meta-package). We calculated the odds ratio (OR) and 95% confidence intervals (CI) for each outcome. A *p*-value < 0.05 was considered statistically significant. Heterogeneity was assessed by visual inspection of the forest plots and measured by *Q* statistic and *I*^2^ statistic. Significant statistical heterogeneity was indicated by *Q* statistic *p*-value less than 0.1 or by *I*^2^ more than 50%. In the case of significant heterogeneity, a random effect model was employed. Otherwise, the fixed effect model was used. Subgroup analysis and sensitivity analysis were used to resolve the heterogeneity.

## Results

### Search strategy results

**Fig. 1:**
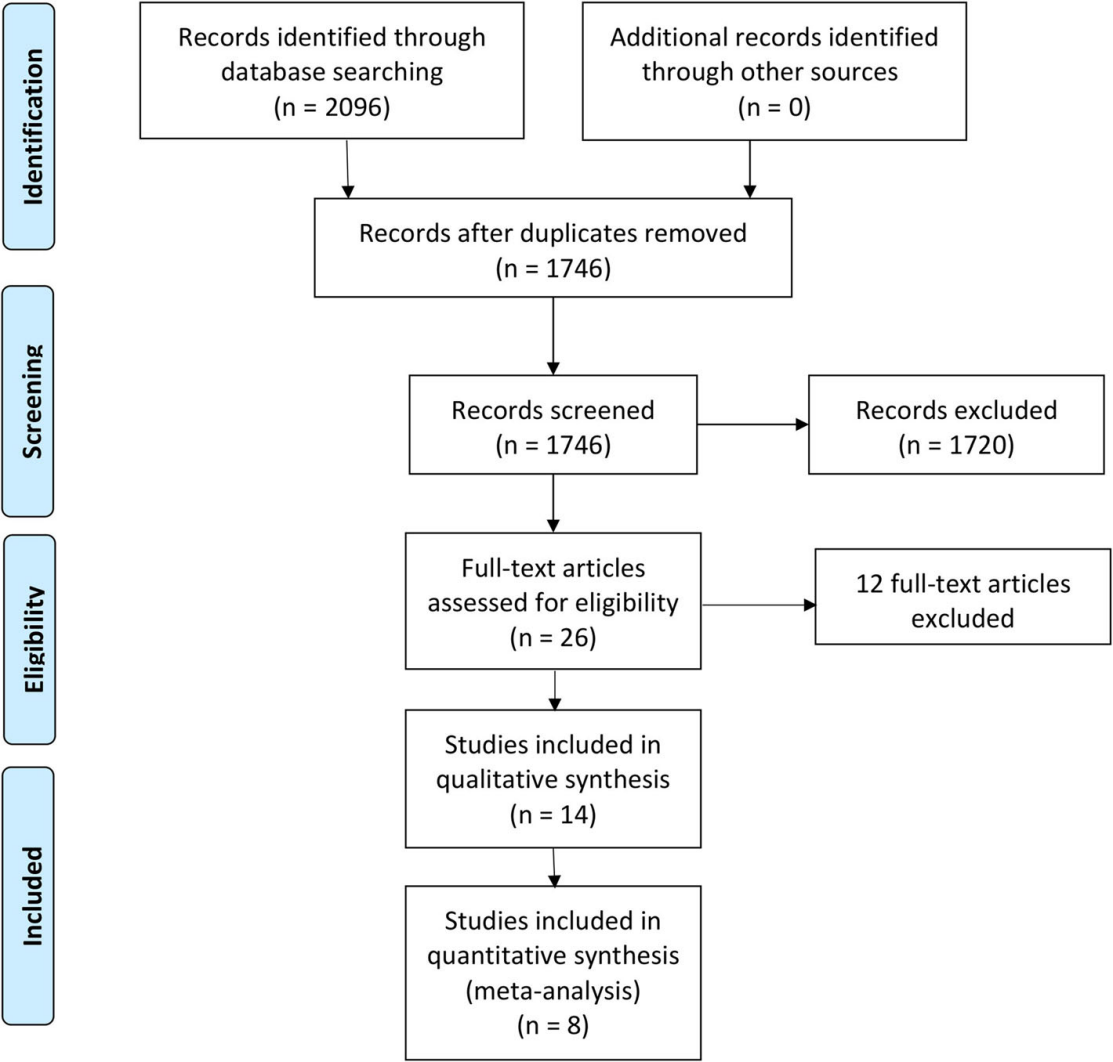
PRISMA flow diagram

Our search retrieved 1746 unique citations. Twenty-six articles were retrieved and screened for eligibility to the systematic review and meta-analysis. Of these, 12 articles were excluded, and 14 studies were included in the systematic review. The PRISMA flow diagram of study selection is shown in Fig. 1.

### Baseline characteristics and risk of bias

Fourteen studies (four RCTs [14, 15, 21, 22] and 10 observational studies [12, 13, 16, 23–29]) enrolling 28,412 patients were included; of them, eight studies were included in the meta-analysis. All articles were published in English from 1989 to 2018. Countries included were the USA (five studies), Taiwan (two studies), Korea (two studies), Canada (one study), China (one study), Colombia (one study), Norway (one study), and Belgium (one study). A summary of the design and baseline characteristics of enrolled patients is presented in Table 1. The included four RCTs reported that the enrolled patients were randomly allocated to different study groups, but only three studies reported how randomization was performed. One used computer-generated random number [14], another study used blinded packaging by the manufacturer [15], and the third generated a random sequence using the Excel software [21]. All RCTs were at low risk of bias in terms of attrition bias and reporting bias, while blinding was unclear in the four RCTs. Observational cohort studies achieved a moderate quality according to the NOS checklist. A summary of risk of bias assessment of RCTs and observational studies is shown in supplementary file 1.

**Table 1:** Summary of included studies

Author, year	Country	Study design	Interventions, No. of patients	Mean age, SD or range (BS vs. control)	Sex, male (BS vs. control)	Study duration	Main findings
Ahn et al. 2018 [21]	Korea	RCT	SB (*n*=25) vs. placebo (*n*=25)	65.5 (16.4)/64.1 (15.4)	18/21	January–December 2015	No difference between SB and placebo in sustained ROSC and survival
Chen et al. 2018 [23]	Taiwan	Population-based cohort study	SB (*n*=1885) vs. control (*n*=3704)	Older than 18 years	1140/2379	1997–2012	Positive association between SB and survival
Kawano et al. 2017 [24]	Columbia	Population-based observational study	SB (*n*=5165) vs. control (*n*=8700)	65 (52–77)/68 (56–80)	3682/5779	2005–2016	SB had less survival
Chung et al. 2015 [25]	China	Retrospective observational study	SB (*n*=74) vs. control (*n*=415)	65.9 (22.7)/72.8 (19.9)	52/237	March–December 2013	SB had a higher percentage of ROSC and survival
Weng et al. 2013 [26]	Taiwan	Retrospective cohort	SB (*n*=30) vs. control (*n*=62)	65/70.5	19/47	January 1–December 2009	SB did not improve rate of ROSC in prolonged (> 15 min) cardiac arrest
Vukmir and Katz 2006 [15]	USA	RCT	SB (*n*=420) vs. control (*n*=372)	67.37 (15.29)/67.16 (14.96)		1994–1998	Overall survival 13.9% (110/792), no difference between groups
Dybvik et al. 1995 [14]	Norway	RCT	SB (*n*=245) vs. saline (*n*=257)	236/266	-	1987–1994	SB therapy had no effect on outcome
Stiell et al. 1995 [28]	Canada	Observational cohort study	BS vs. atropine, Ca, lidocaine, bretylium, procainamide, *n*=529	19 to 98	-	2 years	Logistic regression did not show association between SB and survival
Aufderheide et al. 1992 [16]	USA	Retrospective chart review	SB (*n*=215) vs. No SB (*n*=58)	67 (13)/65 (12)	143/39	1982—1984.	No association between SB and survival
Weaver et al. 1990 [22]	USA	RCT	SB (*n*= 224), lidocaine (*n*=106), epi (*n*=93)	65.6 (12.6)/67.5 (13.2)/66.3 (1.3)	177/89/77	1983–1985	Higher survival with SB infusion
Kim et al. 2016 [13]	Korea	Observational study	SB (*n*=771)	68 (52–77)	375	2008–2013	SB was associated with increased ROSC.
Bar-Joseph et al. 2005 [12]	USA	Retrospective study	SB (*n*=2122)	-	-	1990—1992	Earlier and more frequent use of SB associated with higher ROSC
Bar-Joseph et al. 2002 [27]	USA	Retrospective study	SB (*n*=2915)	-	1955	1990–1992.	SB given in 54% of cases, use increased with ACLS duration
Delooz and Lewi 1989 [29]	Belgium	Retrospective data analysis	SB	-	-	-	SB > 1 mEq/kg associated with poor outcome

*RCT* randomized controlled trial, *SB* sodium bicarbonate, *ROSC* return of spontaneous circulation, *CPR* cardiopulmonary resuscitation, *ACLS* advanced cardiac life support

### Outcomes

#### Survival to discharge

**Fig. 2:**
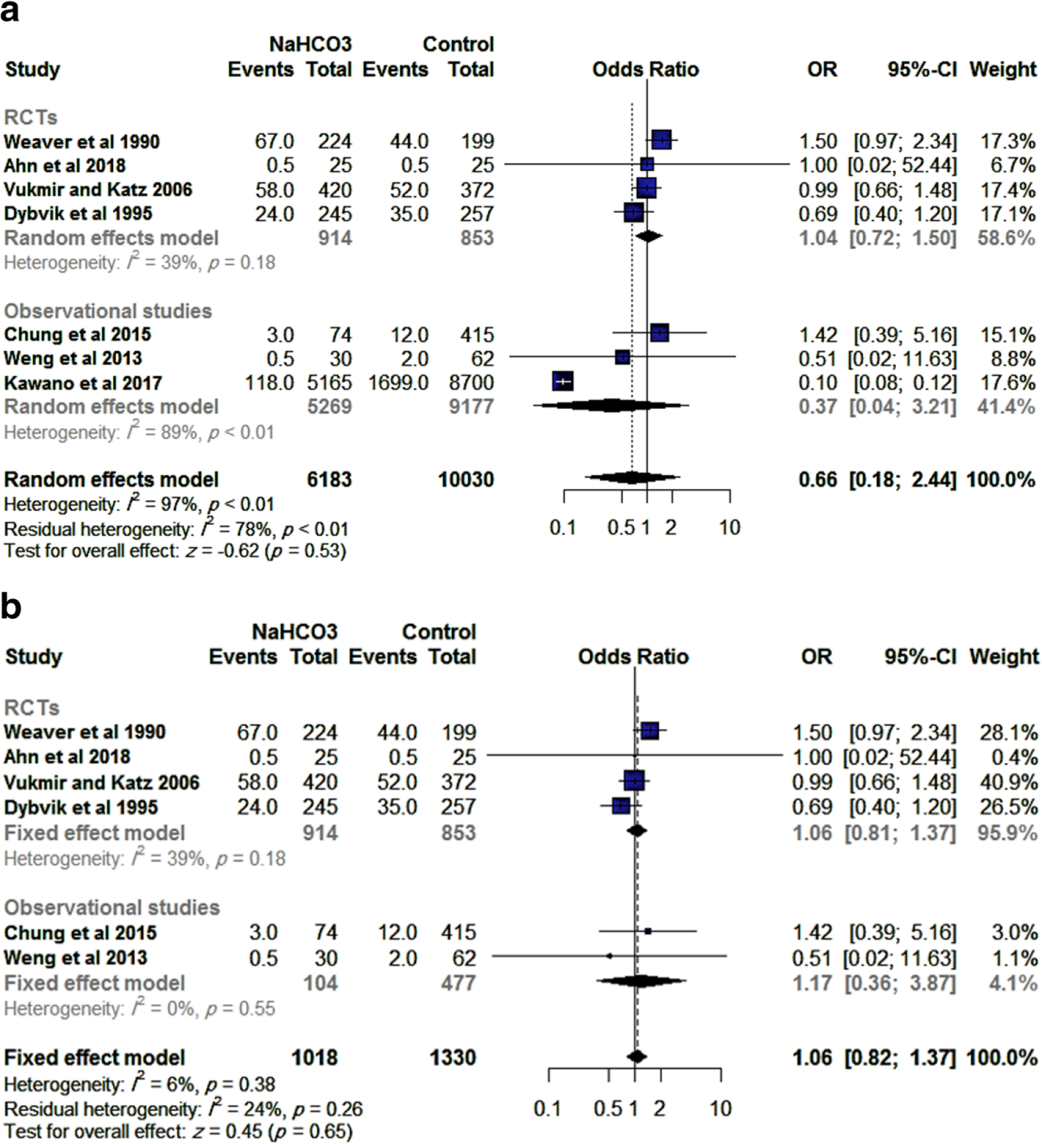
**a** Effect of (SB) administration on survival to discharge. **b** Effect of (SB) administration on survival to discharge after resolution of heterogeneity

Seven studies (four RCTs and three observational studies) reported on the survival rate after discharge from hospital, with a total of 16,213 patients. There was no significant difference between SB or control (OR= 0.66, 95% CI [0.18, 2.44], *p*=0.53), Fig. 2a.

This effect size was consistent with subgroup analysis of RCTs (OR= 1.04, 95% CI [0.72, 1.50], *p*=0.85) and observational studies (OR= 0.37, 95% CI [0.04, 3.21], *p*=0.37). Significant heterogeneity was detected (*I*^2^= 97%; *p*<0.01) which was best resolved by excluding the study by Kawano et al. [24] (OR= 1.06, 95% CI [0.82, 1.37], *p*=0.65, *I*^2^= 6%; *p*=0.38), Fig. 2b.

#### Return of spontaneous circulation (ROSC)

**Fig. 3:**
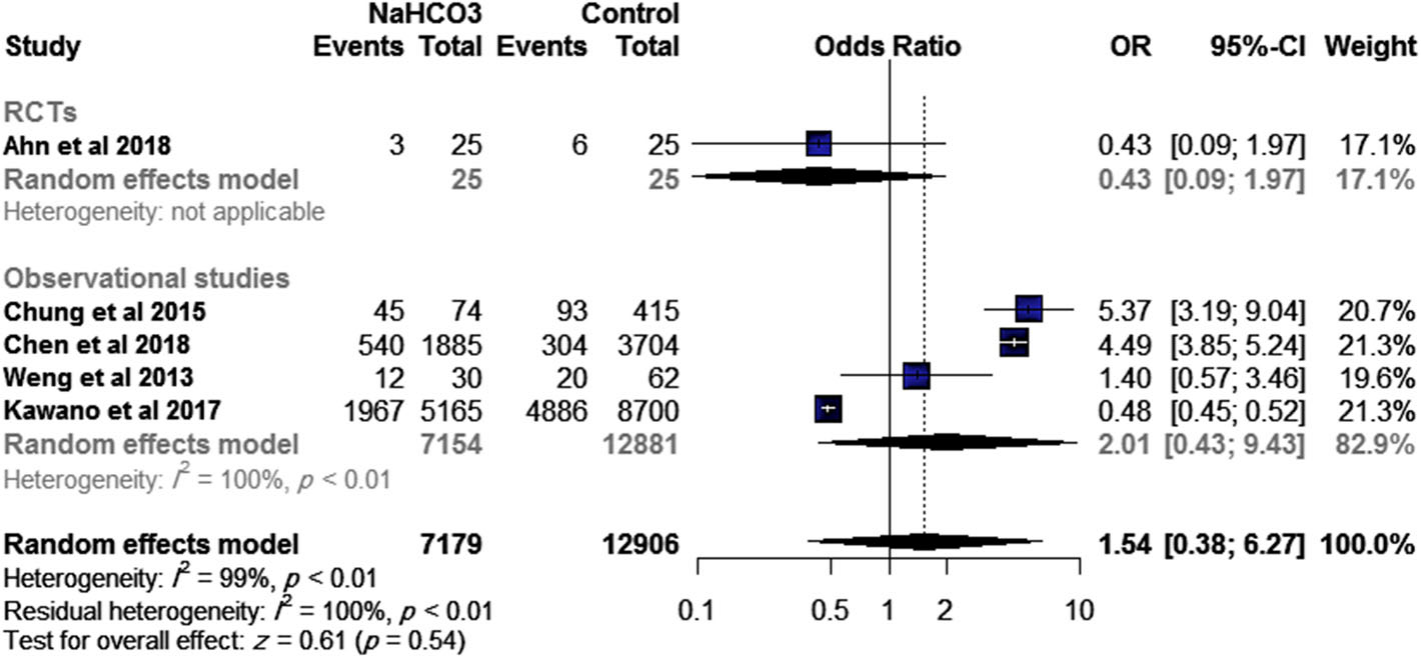
Effect of SB administration on return of spontaneous circulation.

Five studies (one RCT and four observational studies) reported on the rate of ROSC, with a total of 20,085 patients. The overall effect size showed no significant difference between SB and control (OR= 1.54, 95% CI [0.38, 6.27], *p*=0.54), Fig. 3. This effect size was consistent with subgroup analysis according to study design: RCT (OR= 0.43, 95% CI [0.09, 1.97], *p*=0.28) and observational studies (OR= 2.01, 95% CI [0.43, 9.43], *p*=0.38). Significant heterogeneity was detected (*I*^2^= 99%; *p*<0.01) which could not be resolved by sensitivity analysis.

#### Sustained ROSC (> 20 min)

**Fig. 4:**
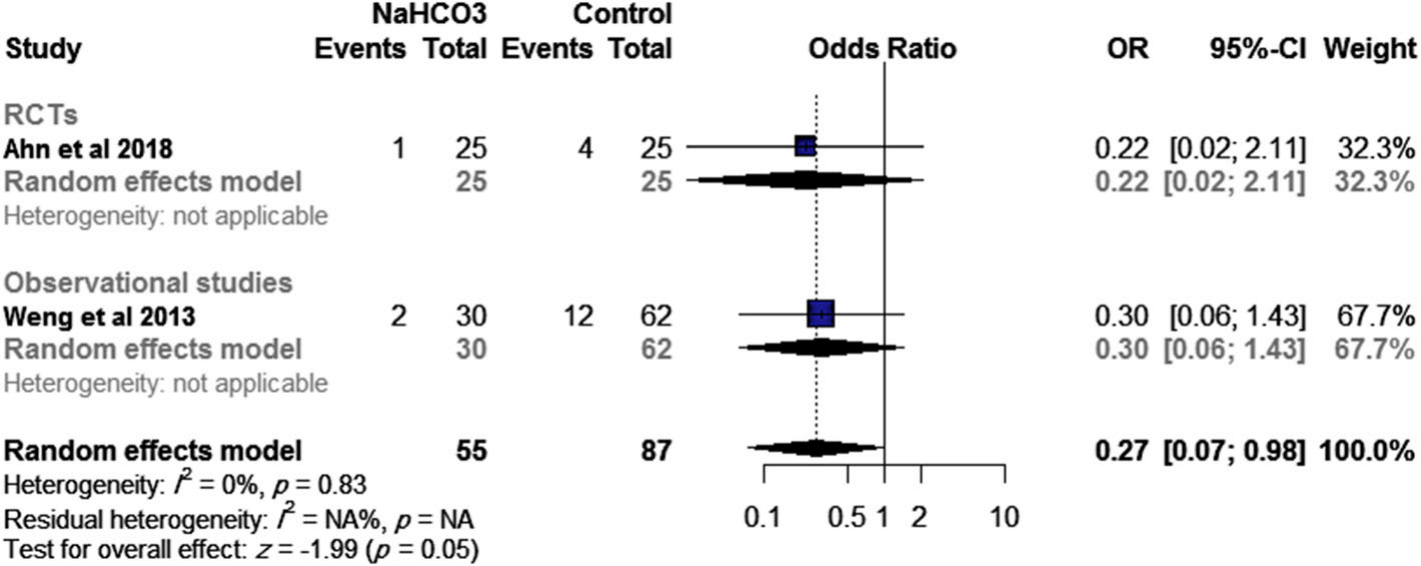
Effect of SB administration on sustained ROSC

The pooled effect size of two studies (one RCT and one observational study, *n*=86 patients) showed that SB was associated with a lower incidence of sustained ROSC than control (OR= 0.27, 95% CI [0.07, 0.98], *p*=0.045), Fig. 4, while this effect size was inconsistent with subgroup analysis of RCT (OR= 0.22, 95% CI [0.02, 2.11], *p*=0.19) and observational study (OR= 0.30, 95% CI [0.06, 1.43], *p*=0.78). No significant heterogeneity was observed (*I*^2^= 0%, *p*=0.83).

### Good neurological outcomes at discharge

**Fig. 5:**
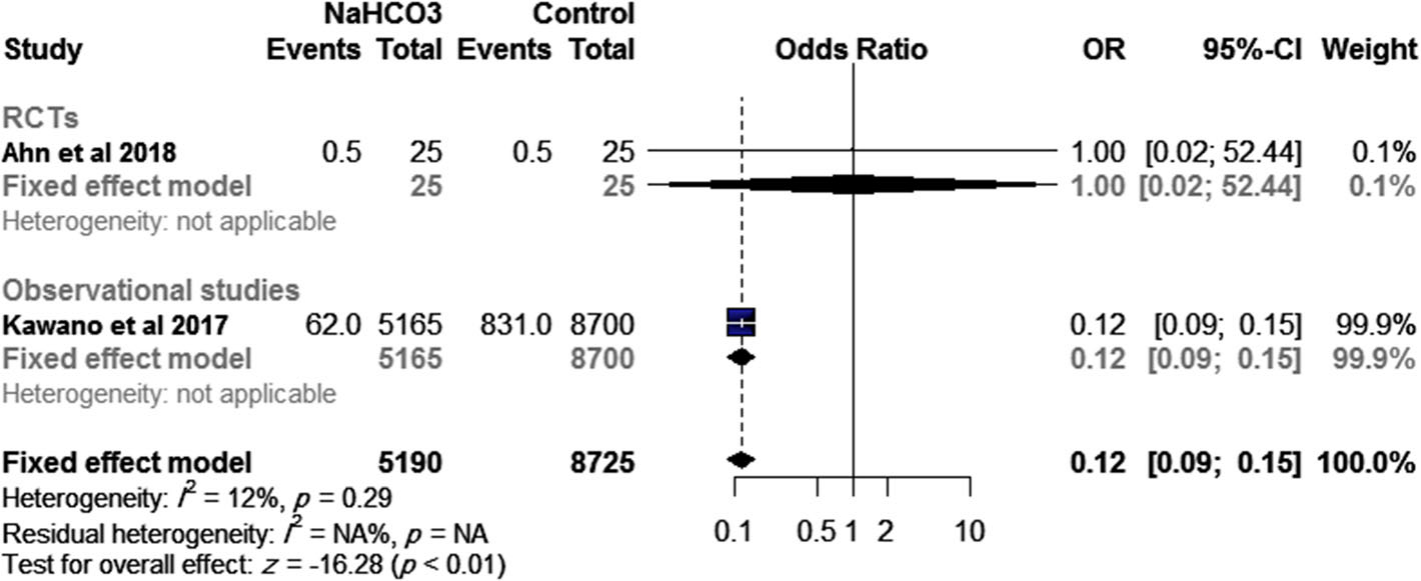
Effect of SB administration on good neurological outcomes at discharge

The pooled effect size of two studies (one RCT and one observational study, *n*=13915) showed that SB was associated with a lower incidence of good neurological outcomes at hospital discharge than control (OR= 0.12, 95% CI [0.09, 0.15], *p*<0.01), Fig. 5. This effect size was consistent with subgroup analysis of the observational study (OR= 0.12, 95% CI [0.09, 0.15], *p*<0.01); however, the RCT did not favor either group (OR= 1.00, 95% CI [0.02, 52.44], *p*=1.00). No significant heterogeneity was observed (*I*^2^= 12%, *p*=0.29).

### Narrative review of other studies

Kim et al. conducted an observational study in 2016 to assess the relationship between BS and incidence of ROSC and found that SB and its cumulative dose were significantly related to a higher incidence of ROSC within 20 min, and this relation kept true after adjustment with multivariable conditional logistic regression analysis [13]. Bar-Joseph et al. in 2002 conducted a retrospective study based on brain resuscitation clinical trial and concluded that, when bicarbonate was used, it was probably used late, and suggested that, because of development of severe metabolic acidosis, bicarbonate administration should start early [27]. Another study by Bar-Joseph et al. in 2005 reported a survival rate of 33.5% and 25.7% in high SB and low SB user sites, respectively. Regarding hospital discharge rate, a prevalence of 5.3% was reported in the high SB user sites compared to 3% in the “low SB user” sites. In addition, 5.3% of high SB users achieved a favorable neurological outcome compared to 2.1% in the low SB user sites [12].

Stiell et al. conducted an observational cohort study in 1995 and reported that SB did not have a significant association with survival. However, the authors noted that the timing of drug administration could be an important factor [28]. Aufderheide et al. reviewed 619 cardiac arrest patients and reported that the survival rate was comparable between SB and control. However, base changes were significantly improved in the 15- to 20-min CPR time interval [16]. Delooz and Lewi conducted a retrospective data analysis in 1989 and reported that SB > 1 mEq/kg was associated with poor outcome [29].

## Discussion

Our results showed that SB was not superior to the control group in terms of survival to discharge and return of spontaneous circulation. Moreover, SB administration was associated with lower rates of sustained ROSC and good neurological outcomes than the control group. These findings discourage the use of SB in OHCA patients.

The onset of SB use in CPR originated in the 1960s from physiological sense, assuming that the resulting acidosis from ischemic injury would prevent successful resuscitation [30]. However, Levine has concluded that in some situations, acidosis may actually be protective [31]. Moreover, several later studies failed to show significant benefits for SB in cardiac arrest [16, 28], along with the potential adverse effects as reduction of systemic vascular resistance, compromised coronary flow, hypernatremia, and hyperosmolarity. Therefore, SB administration was classified as class III status (inappropriate, without scientific evidence of efficacy) for CPR [32].

In 2010, the American Heart Association released resuscitation guidelines that recommended against the routine use of SB in CPR, except in patients with metabolic acidosis, hyperkalemia, or their arrest is caused by tricyclic antidepressant overdose [10]. In patients with OHCA, CPR is usually protracted, and the occurrence of metabolic acidosis is more frequent than in-hospital cardiac arrest. These guidelines were not updated in the 2015 version [11]. However, the adherence to these guidelines is not optimal. Authors of the BRCT-III trial showed that clinicians do not consider the pre-ALS hypoxia time (the major contributor to metabolic acidosis in cardiac arrest) when deciding to administer SB. They called upon guideline developers to emphasize the importance of pre-ALS hypoxia time and be more specific in determining protracted CPR efforts [27].

Despite this progress, the issue remains controversial. Some recent studies were published on the topic, advocating or negating the benefit of SB administration in OHCA [13, 21, 23, 24], while a recently published systematic review and meta-analysis included 6 observational studies that showed the same results where SB use was not associated with improvement in ROSC or survival-to-discharge rates in cardiac resuscitation [33]. The results of this analysis extend the current guidelines that SB administration in OHCA is not superior to control in terms of efficacy. To investigate whether the prevalence of retrospective studies affects the results of the analysis, we performed a subgroup analysis based on the study design. The results of the subgroup analyses were consistent with the overall analysis in most outcomes, except for sustained ROSC and achievement of good neurological outcomes. Moderate to significant heterogeneity was observed in most outcomes. This may be related to the different SB administration protocols.

## Limitations

Some limitations to this meta-analysis should be highlighted. First, the majority of the included studies were observational in nature, which is liable to potential confounders. Second, moderate to significant heterogeneity was observed in most outcomes. This may be related to the different SB administration protocols with different timing of administration and routes. Several studies suggested that the protocol and earlier timing of SB administration factor in the resulting efficacy; however, the optimal protocol for SB administration in cardiac arrest remains unconfirmed [27, 28]. Similarly, Chung et al. noted that the beneficial effects of SB were dose-dependent; higher doses (> 100 ml) were associated with higher rates of ROSC [25].

## Conclusion

The results of this meta-analysis—based on the published evidence—showed that the use of SB was not associated with improvement in survival to discharge, rate of ROSC, rate of sustained ROSC, and good neurological outcomes at hospital discharge. Until further evidence and characterization of the potentially useful dose and timing of SB administration are available, clinicians and emergency responders are not advised to routinely administer SB in OHCA patients.

## Supplementary Information

**Additional file 1.**

## Data Availability

All data generated or analyzed during this study are included in this published article and its supplementary information files.

## Abbreviations

ALS: Advanced life support
OHCA: Out-of-hospital cardiac arrest
ROSC: Return of spontaneous circulation
CPR: Cardiopulmonary resuscitation
SB: Sodium bicarbonate

## References

[1] Neumar RW, Otto CW, Link MS, Kronick SL, Shuster M, Callaway CW, Kudenchuk PJ, Ornato JP, McNally B, Silvers SM, Passman RS, White RD, Hess EP, Tang W, Davis D, Sinz E, Morrison LJ (2010). Part 8: adult advanced cardiovascular life support: 2010 American Heart Association Guidelines for Cardiopulmonary Resuscitation and Emergency Cardiovascular Care. Circulation.

[2] Roger VL, Go AS, Lloyd-Jones DM, Adams RJ, Berry JD, Brown TM, Carnethon MR, Dai S, de Simone G, Ford ES, Fox CS, Fullerton HJ, Gillespie C, Greenlund KJ, Hailpern SM, Heit JA, Ho PM, Howard VJ, Kissela BM, Kittner SJ, Lackland DT, Lichtman JH, Lisabeth LD, Makuc DM, Marcus GM, Marelli A, Matchar DB, McDermott MM, Meigs JB, Moy CS, Mozaffarian D, Mussolino ME, Nichol G, Paynter NP, Rosamond WD, Sorlie PD, Stafford RS, Turan TN, Turner MB, Wong ND, Wylie-Rosett J, American Heart Association Statistics Committee and Stroke Statistics Subcommittee (2011). Heart disease and stroke statistics--2011 update: a report from the American Heart Association. Circulation.

[3] McNally B, Robb R, Mehta M, Vellano K, Valderrama AL, Yoon PW, Sasson C, Crouch A, Perez AB, Merritt R, Kellermann A, Centers for Disease Control and Prevention (2011). Out-of-hospital cardiac arrest surveillance --- Cardiac Arrest Registry to Enhance Survival (CARES), United States, October 1, 2005--December 31, 2010. MMWR Surveill Summ.

[4] Standards for cardiopulmonary resuscitation (CPR) and emergency cardiac care (ECC). 3 (1974). Advanced life support. JAMA.

[5] Batenhorst RL, Clifton GD, Booth DC, Hendrickson NM, Ryberg ML (1985). Evaluation of 516 cardiopulmonary resuscitation attempts. Am J Hosp Pharm.

[6] Standards and guidelines for cardiopulmonary resuscitation (CPR) and emergency cardiac care (ECC) (1986). JAMA.

[7] Guidelines for cardiopulmonary resuscitation and emergency cardiac care. Emergency Cardiac Care Committee and Subcommittees, American Heart Association. Part III (1992). Adult advanced cardiac life support. JAMA.

[8] Guidelines 2000 for Cardiopulmonary Resuscitation and Emergency Cardiovascular Care. Part 6: advanced cardiovascular life support: section 6: pharmacology II: agents to optimize cardiac output and blood pressure (2000). The American Heart Association in collabor. Circulation.

[9] Kette F, Weil MH, Gazmuri RJ (1991). Buffer solutions may compromise cardiac resuscitation by reducing coronary perfusion presssure. JAMA.

[10] Vanden Hoek TL, Morrison LJ, Shuster M, Donnino M, Sinz E, Lavonas EJ, Jeejeebhoy FM, Gabrielli A (2010). Part 12: cardiac arrest in special situations: 2010 American Heart Association Guidelines for cardiopulmonary resuscitation and emergency cardiovascular care. Circulation.

[11] Lavonas EJ, Drennan IR, Gabrielli A, Heffner AC, Hoyte CO, Orkin AM, Sawyer KN, Donnino MW (2015). Part 10: special circumstances of resuscitation. Circulation.

[12] Bar-Joseph G, Abramson NS, Kelsey SF, Mashiach T, Craig MT, Safar P, Brain Resuscitation Clinical Trial III (BRCT III) Study Group (2005). Improved resuscitation outcome in emergency medical systems with increased usage of sodium bicarbonate during cardiopulmonary resuscitation. Acta Anaesthesiol Scand.

[13] Kim J, Kim K, Park J, Jo YH, Lee JH, Hwang JE, Ha C, Ko Y, Jung E (2016). Sodium bicarbonate administration during ongoing resuscitation is associated with increased return of spontaneous circulation. Am J Emerg Med.

[14] Dybvik T, Strand T, Steen PA (1995). Buffer therapy during out-of-hospital cardiopulmonary resuscitation. Resuscitation.

[15] Vukmir RB, Katz L, Sodium Bicarbonate Study Group (2006). Sodium bicarbonate improves outcome in prolonged prehospital cardiac arrest. Am J Emerg Med.

[16] Aufderheide TP, Martin DR, Olson DW, Aprahamian C, Woo JW, Hendley GE, Hargarten KM, Thompson B (1992). Prehospital bicarbonate use in cardiac arrest: a 3-year experience. Am J Emerg Med.

[17] Higgins JP, Green S (2008). Cochrane Handbook for Systematic Reviews of intervemntions.

[18] Moher D, Liberati A, Tetzlaff J, Altman DG (2009). Preferred reporting items for systematic reviews and meta-analyses: the PRISMA statement. PLoS Med.

[19] JPT H, Altman DG, Gotzsche PC, Juni P, Moher D, Oxman AD, Savovic J, Schulz KF, Weeks L, JAC S, Cochrane Bias Methods Group, Cochrane Statistical Methods Group (2011). The Cochrane Collaboration’s tool for assessing risk of bias in randomised trials. BMJ.

[20] Stang A (2010). Critical evaluation of the Newcastle-Ottawa scale for the assessment of the quality of nonrandomized studies in meta-analyses. Eur J Epidemiol.

[21] Ahn S, Kim Y-J, Sohn CH, Seo DW, Lim KS, Donnino MW, Kim WY (2018). Sodium bicarbonate on severe metabolic acidosis during prolonged cardiopulmonary resuscitation: a double-blind, randomized, placebo-controlled pilot study. J Thorac Dis.

[22] Weaver WD, Fahrenbruch CE, Johnson DD, Hallstrom AP, Cobb LA, Copass MK (1990). Effect of epinephrine and lidocaine therapy on outcome after cardiac arrest due to ventricular fibrillation. Circulation.

[23] Chen Y-C, Hung M-S, Liu C-Y, Hsiao C-T, Yang Y-H (2018). The association of emergency department administration of sodium bicarbonate after out of hospital cardiac arrest with outcomes. Am J Emerg Med.

[24] Kawano T, Grunau B, Scheuermeyer FX, Gibo K, Dick W, Fordyce CB, Dorian P, Stenstrom R, Straight R, Christenson J (2017). Prehospital sodium bicarbonate use could worsen long term survival with favorable neurological recovery among patients with out-of-hospital cardiac arrest. Resuscitation.

[25] Chung C, Lui C, Tsui K (2015). Role of sodium bicarbonate in resuscitation of out-of-hospital cardiac arrest. Hong Kong J Emerg Med.

[26] Weng Y-M, Wu S-H, Li W-C, Kuo C-W, Chen S-Y, Chen J-C (2013). The effects of sodium bicarbonate during prolonged cardiopulmonary resuscitation. Am J Emerg Med.

[27] Bar-Joseph G, Abramson NS, Jansen Williams L, Kelsey SF, Mashiach T, Craig MT, Safar P, Brain Resuscitation Clinical Trial III (BRCT III) Study Group (2002). Clinical use of sodium bicarbonate during cardiopulmonary resuscitation--is it used sensibly?. Resuscitation.

[28] Stiell IG, Wells GA, Hebert PC, Laupacis A, Weitzman BN (1995). Association of drug therapy with survival in cardiac arrest: limited role of advanced cardiac life support drugs. Acad Emerg Med.

[29] Delooz HH, Lewi PJ (1989). Are inter-center differences in EMS-management and sodium-bicarbonate administration important for the outcome of CPR? The Cerebral Resuscitation Study Group. Resuscitation.

[30] Gazmuri RJ (1999). Buffer treatment for cardiac resuscitation: putting the cart before the horse?. Crit Care Med.

[31] Levine RL (1993). Ischemia: from acidosis to oxidation. FASEB J.

[32] Christenson JM, Solimano AJ, Williams J, Connolly B, Monik L, Erb-Campbell H, McGonigle L (1993). The new American Heart Association guidelines for cardiopulmonary resuscitation and emergency cardiac care: presented by the Emergency Cardiac Care Subcommittee of the Heart and Stroke Foundation of Canada. CMAJ.

[33] Wu KH, Chang CY, Chen YC, Chang CP, Hsiao CT, Weng HH (2020). Effectiveness of sodium bicarbonate administration on mortality in cardiac arrest patients: a systematic review and meta-analysis. J Emerg Med.

