# Supplementary material for: Use of sodium bicarbonate in out-of-hospital cardiac arrest: a systematic review and meta-analysis
Source: Int J Emerg Med. 2021 Apr 13;14:21. doi: 10.1186/s12245-021-00344-x (PMC8042972; doi:10.1186/s12245-021-00344-x)
Supplement: Supplementary file 1 — Additional file 1. [file 12245_2021_344_MOESM1_ESM.docx]

**Supplementary file 1**

Risk of bias assessment of the included randomized controlled trial.

| **Ahn et al. 2018** | **Risk of Bias** | **Reason/Quotations** |
| --- | --- | --- |
| Random sequence generation (selection bias) | Low risk | A research associate generated a random sequence using Excel software. Patients fulfilling the study inclusion criteria were randomly assigned (1:1). |
| Allocation concealment (selection bias) | Unclear |  |
| Blinding of participants and personnel (performance bias) | Unclear |  |
| Blinding of outcome assessment (detection bias) | Unclear |  |
| Incomplete outcome data (attrition bias) | Low risk | All patients who were randomized and received study drug were included in the study analysis. |
| Selective reporting (reporting bias) | Low risk | All major outcomes were reported. |
| Other bias | Unclear |  |
| **Vukmir and Katz 2006** |  |  |
| Random sequence generation (selection bias) | Low risk | Randomization was accomplished by blinded packaging by the manufacturer (Abbott Laboratories), and every patient received study intervention. |
| Allocation concealment (selection bias) | Unclear |  |
| Blinding of participants and personnel (performance bias) | Unclear |  |
| Blinding of outcome assessment (detection bias) | Unclear |  |
| Incomplete outcome data (attrition bias) | Low risk | All patients who were randomized and received study drug were included in the study analysis. |
| Selective reporting (reporting bias) | Low risk | All major outcomes were reported. |
| Other bias | Unclear |  |
| **Dybvik et al. 1995** |  |  |
| Random sequence generation (selection bias) | Low risk | A computer-generated random number list was performed in blocks of 10 patients with equal probability for each permutation within each block. |
| Allocation concealment (selection bias) | Unclear |  |
| Blinding of participants and personnel (performance bias) | Unclear |  |
| Blinding of outcome assessment (detection bias) | Unclear |  |
| Incomplete outcome data (attrition bias) | Low risk | All patients who were randomized and received study drug were included in the study analysis. |
| Selective reporting (reporting bias) | Low risk | All major outcomes were reported. |
| Other bias | Unclear |  |
| **Weaver et al. 1990** |  |  |
| Random sequence generation (selection bias) | Unclear |  |
| Allocation concealment (selection bias) | Unclear |  |
| Blinding of participants and personnel (performance bias) | High risk | Open label |
| Blinding of outcome assessment (detection bias) | Unclear |  |
| Incomplete outcome data (attrition bias) | Low risk | Where appropriate, the results were  analyzed on the basis of intention to treat and actual treatment received. |
| Selective reporting (reporting bias) | Low risk | All major outcomes were reported. |
| Other bias | Unclear |  |

**Methodological quality of the included observational study based on the Newcastle Ottawa scale for assessing the quality of epidemiological studies.**

| **Study** | **Selection** | | | | **Comparability** | **Exposure** | | | **Total Score** |
| --- | --- | --- | --- | --- | --- | --- | --- | --- | --- |
|  | **Representativeness**  **of the exposed**  **cohort** | **Selection of the non-exposed cohort** | **Ascertainment of exposure^1^** | **Outcome was not present at start of study^2^** | **Control for  2 important factors^3.4^** | **Assessment of outcome** | **Follow-up long enough** | **Adequacy of**  **follow-up of**  **cohort^7^** |  |
| Chen et al. 2018 | * | * | * | - | * | * | * | * | 7 |
| Kawano et al. 2017 | * | * | * | - | * | * | * | * | 7 |
| Chung et al 2015 | * | * | - | - | * | * | * | * | 6 |
| Weng et al. 2013 | * | * | * | - | * | * | * | * | 7 |
| Stiell et al. 1995 | * | * |  | - | * | * | * | * | 6 |
| Aufderheide et al. 1992 | * | * | * | - | * | * | * | * | 7 |
| Kim et al. 2016 | * | - | * | * | * | * | * | * | 7 |
| Bar-Joseph et al. 2002 | * | - | * | - | * | * | * | * | 6 |
| Bar-Joseph et al. 2005 | * | - | * | - | * | * | * | * | 6 |
| Delooz and Lewi 1989 | * | - | - | - | * | * | * | * | 5 |

^1^ If the exposure data was obtained from prescription database or medical record, a point was assigned.

^2^ If the study design is prospective study, a point was assigned.

^3^ If adjusted for age, a point was assigned.
^4^ If adjusted for any other additional factors, a point was assigned.
^5^ If the completeness of follow-up was 80% or more, a point was assigned.

The methodological quality of the included case reports and case series.

| Study | Selection | Ascertainment | | Causality | | | | Reporting | Total score |
| --- | --- | --- | --- | --- | --- | --- | --- | --- | --- |
|  | Patient(s) represent(s) the whole experience of the investigator | The exposure adequately ascertained | The outcome adequately ascertained | Alternative causes that may explain the observation ruled out | There was a challenge/rechallenge phenomenon | There was a dose-response effect | Follow-up long enough | Case(s) described with sufficient details |  |
| Nobrega et al. 2017 | * | * | * | * | - | * | * | * | 7 |
| Sheehy et al. 2017 | * | * | * | - | - | * | * | * | 6 |
| Chu et al. 2013 | * | * | * | * | - | * | * | * | 7 |
| Palm et al. 2018 | * | * | * | - | - | * | * | * | 6 |
| Hassel et al. 2017 | * | * | * | - | - | * | * | * | 6 |
| Tawfic et al. 2014 | * | * | * | - | - | * | * | * | 6 |
| Zempsky et al. 2010 | * | * | * | - | - | * | * | * | 6 |
| Gimovsky et al 2017 | * | * | * | - | - | * | * | * | 6 |
| Jennings et al. 2013 | * | * | * | - |  | * | * | * | 6 |
| Uprety et al. 2013 | * | * | * | - | - | * | * | * | 6 |
| Meals et al. 2011 | * | * | * | - | - | * | * | * | 6 |
| Kerr et al. 2011 | * | * | * | - | - | * | * | * | 6 |
